# Morphologic Variation of Liver Associated With Hourglass Stomach: Case Report and Literature Review

**DOI:** 10.7759/cureus.23022

**Published:** 2022-03-10

**Authors:** Sushma R Poleneni, Prashant Munjamkar, Mrudula Chandrupatla

**Affiliations:** 1 Anatomy, Employees’ State Insurance Corporation Medical College and Hospital, Hyderabad, IND; 2 Anatomy, Shree Shankaracharya Institute of Medical Sciences, Bhilai, IND; 3 Anatomy, All India Institute of Medical Sciences, Bibinagar, Bibinagar, IND

**Keywords:** diagnostic error, constriction, stomach, accessory lobe, liver, anatomy

## Abstract

Ectopic liver tissue is a rare developmental abnormality, and its association with hourglass constriction of the stomach is undocumented to date. This case report describes the discovery of morphologic variations in the liver of an adult female cadaver during routine dissection. The variations include a small, pedunculated, club-shaped accessory lobe covered by a glistening fibrous capsule connecting it to the gallbladder wall, with vessels radiating into the lobe. Two additional lobes were present, one attached to the right upper margin of the caudate lobe, overlapping the inferior vena cava, and another near the quadrate lobe. The right lobe had an abnormal shape with multiple incomplete fissures and furrows. The left lobe was hypoplastic with an elongated end, resembling a lingular process. Further dissection revealed a prominent fibrous band on the posterior surface of the stomach, which continued anteriorly, giving it an hourglass appearance. Knowledge of such variations helps surgeons and radiologists rule out related abnormalities.

## Introduction

Anatomically, the liver is under the right dome of the diaphragm. It is divided into right and left lobes by the attached falciform ligament along the superior anterior surface and posteriorly by the ligamentum teres and ligamentum venosus. Embryonic development of the liver is complex, with the early embryonic organ being multilobular and development of accessory lobes being rare [1]. An increase in liver lobation can resemble that of the lower mammals. Accessory lobes in humans are typically found incidentally but may also be a result of neoplasia or a compressive effect. These accessory lobes have increased potential for developing hepatocellular carcinoma in the absence of malignancy in the mother liver [2]. The most frequent presentation of an accessory lobe is the Riedel lobe with hypertrophy of segments V and VI.

The stomach is the most dilated part of the gastrointestinal tract and is present between the esophagus and duodenum: the shape of the stomach changes depending on the contents of the sac. Clinically, the stomach is divided into three types: sthenic, hypersthenic, and hyposthenic. The sthenic type is J-shaped and is considered typical. The hypersthenic type or “steer-horn stomach” is more obliquely oriented and prone to duodenal ulcers, whereas the hyposthenic type is more vertically oriented and prone to gastric ulcers [3]. An “hourglass stomach” is a variant in which the stomach muscles contract and change the shape from normal to hourglass with two chambers. Another unique variety is “cascade stomach.” The upper chamber loses peristaltic control, and only after filling the upper/ posterior chamber do the stomach contents trickle into the lower chamber [4].

## Case presentation

This case report describes the morphologic variations in an adult female cadaver (age 65 years) during routine dissection. The steps for the dissection were performed according to Cunningham’s Manual of Practical Anatomy [5].

First variation

**Figure 1:**
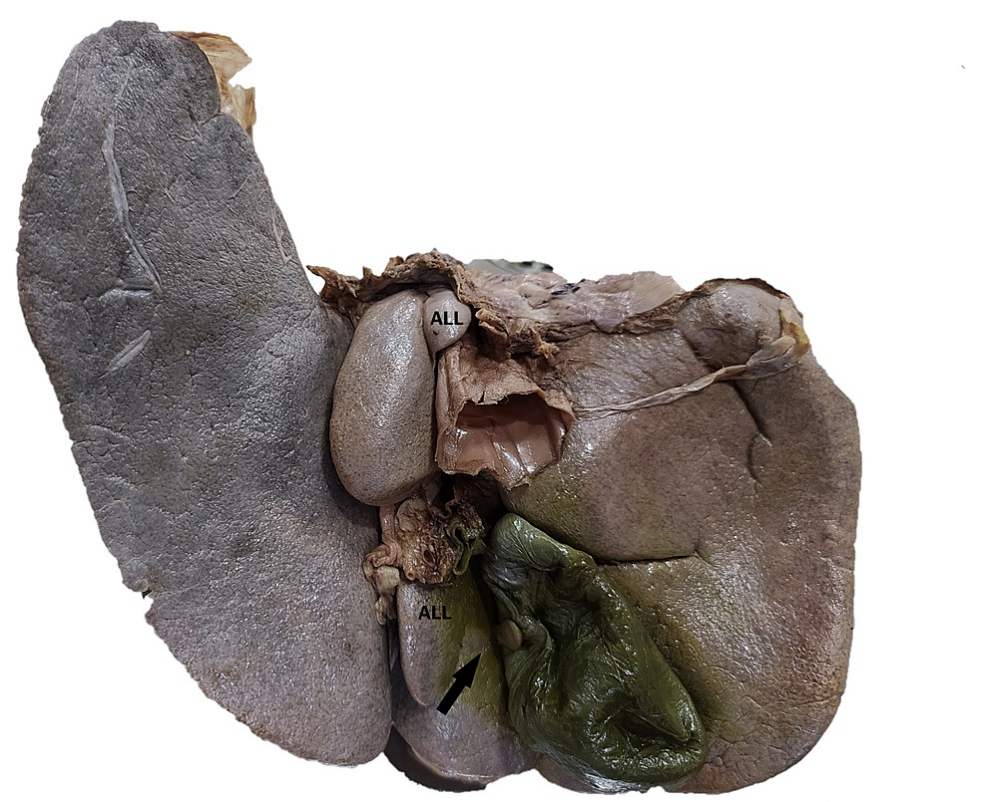
Posteroinferior surface of the liver showing a mini-accessory lobe (black arrow). ALL = Accessory liver lobe.

**Figure 2:**
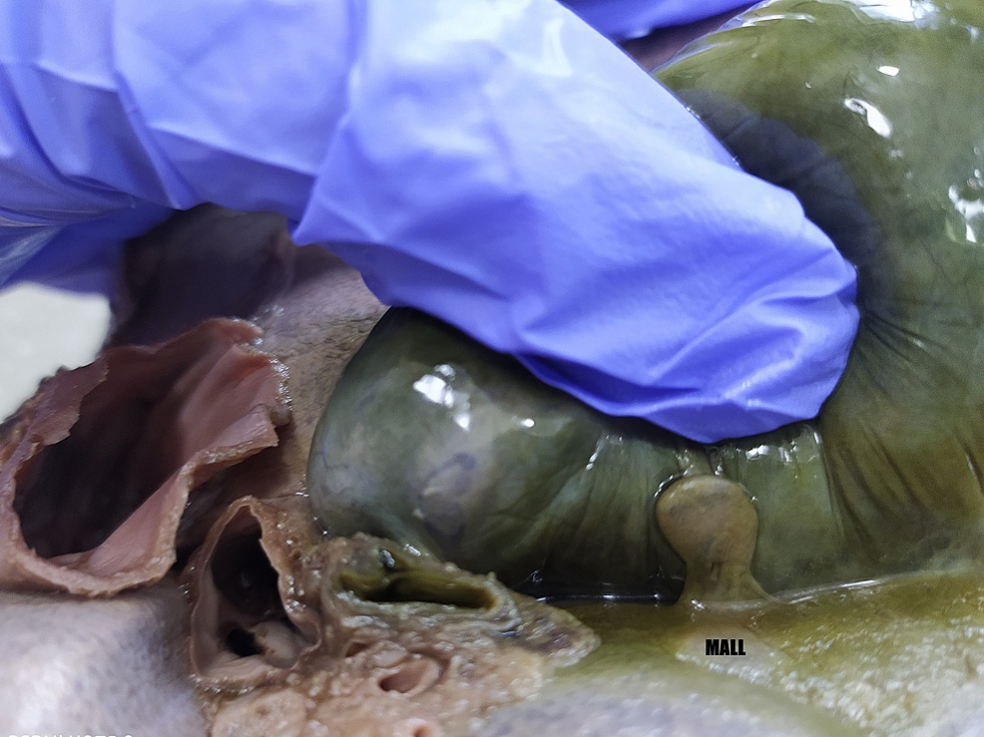
Pedunculated mini-accessary lobe of the liver attached to the gallbladder. MALL = mini-accessory liver lobe.

The right lobe had multiple fissures and furrows that partially divided it into an incomplete lobe. The caudate lobe was large, with an accessory lobe attached to its upper right margin, which presented as a thin extension overlapping the inferior vena cava. The quadrate lobe was small, with an incomplete fissure dividing it from the right lobe (Figure 1). A pedunculated, club-shaped, mini-accessory lobe (1.1 × 0.6 × 0.2 cm) was present, arising from the inferior surface of the right lobe of the liver, adjacent to the right margin of the cystic fossa. The accessory lobe was covered by a fibrous capsule firmly adherent to the gallbladder wall (Figure 2). In the peduncle, vessels passed through and supplied the accessory lobe. The left lobe was hypoplastic with a tongue-like lingular projection extending into the left hypochondrium and epigastrium. There were two incomplete furrows on the anterosuperior surface, partially dividing the right lobe and forming an incomplete apical lobule.

**Figure 3:**
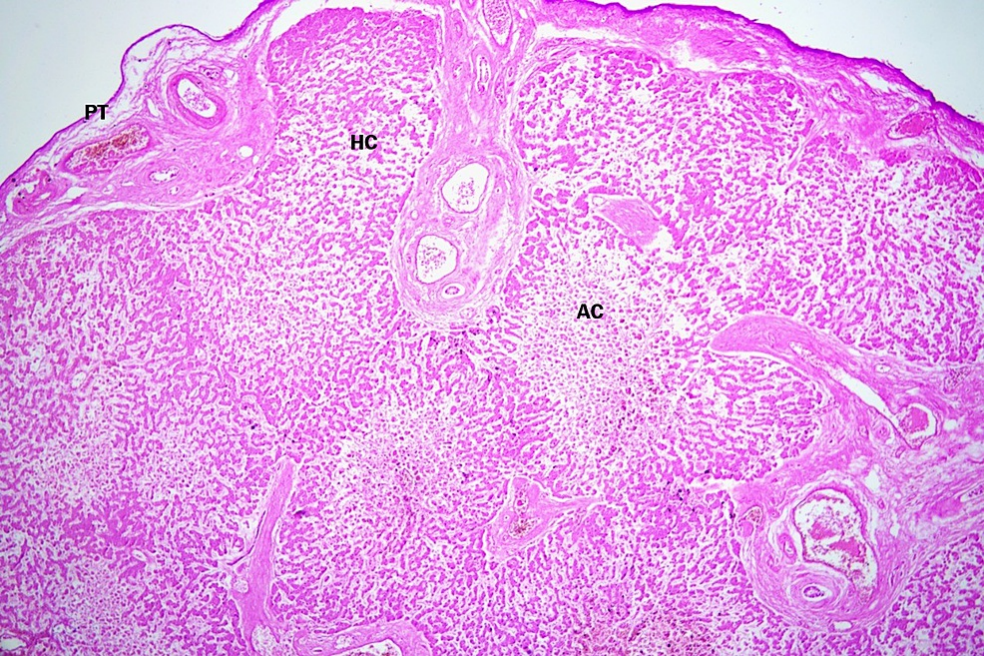
Photomicrograph of the accessory lobe showing normal hepatocytes mixed with apoptotic cells. Fibrous capsule with portal triad structures (hematoxylin and eosin staining, 10 × 10 magnification). AC = apoptotic cells; HC = hepatocytes; PT = portal triad.

Histologic examination of the accessory lobe revealed normal hepatocytes with apoptotic cells interspersed between them in that section. The accessory lobe’s viability depends on the hepatic artery, hepatic vein, portal vein, and bile duct. In this section, vessels and a duct were present in the fibrous capsule surrounding the accessory lobe (Figure 3). The typical hepatic lobule architecture was absent in that section of the slide because of a central vein surrounded by hepatocytes.

Second variation

**Figure 4:**
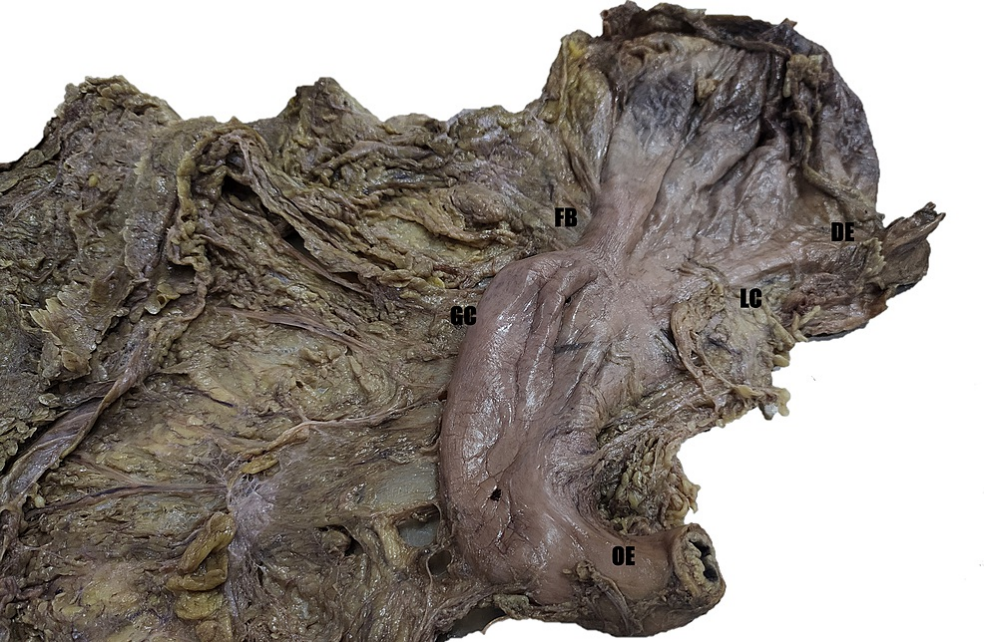
The posterior aspect of the stomach shows a fibrous band and an hourglass stomach shape. DE = duodenal end; FB = fibrous end; GC = greater curvature; LC = lesser curvature; OE = esophageal end.

During the stomach examination in the same cadaver, further dissection showed an atypical appearance with a prominent muscular band on the posterior surface that continued on the anterior surface, constricting the stomach into an hourglass appearance (Figure 4). The distance of constriction from the pylorus along the greater curvature was 7 cm and along the lesser curvature was 5.5 cm. The constriction divided the stomach into an upper and lower pouch. On further dissection, the interior of the mucosa was normal, with multiple rugae extending along the inner surface.

## Discussion

Variations of liver morphology are rare and correspond to developmental anomalies during embryogenesis; these variations are typically incidental findings detected during imaging [6]. The most common anomalies of the liver are variations in the shape and number of lobules, whereas the presence of an accessory lobe is a rare finding. The liver primordium as a hepatic bud appears in the middle of the third week as an endodermal outgrowth of the distal end of the foregut. The cranial end forms the larger pars hepatica, and the smaller caudal end forms the pars cystica. The pars hepatica forms the right and left lobes of the liver, which enlarge and invade the septum transversum. The pars cystica form the gallbladder and cystic duct [7]. Cells of the pars hepatica are arranged as sheets and form the radiating cords of the hepatic lobule. The umbilical and vitelline veins interspersed in the septum transversum dissolve and form spaces that later form sinusoids. The mesoderm between the liver and ventral abdominal wall forms the falciform ligament and between the liver and foregut forms the lesser omentum [8].

In addition to variations in liver morphology, an hourglass constriction of the stomach was observed in the same cadaver. Embryologically, the stomach appears as a fusiform dilatation of the foregut in the fourth week, and further changes in position and appearance occur during consequent weeks. The stomach rotates around the longitudinal axis and anteroposterior axis in a 90°clockwise direction, with its left side facing anteriorly and right side facing posteriorly. The rotation occurs due to an enlarging liver and the developing omental bursa. The liver displaces the freely movable cranial end of the stomach to the left. In contrast, the caudal end is anchored by short ventral mesentery and is responsible for the slanting position of the stomach. Any anomaly during this rotation can result in variations in the stomach morphology.

Hepatic abnormalities are one of two types: either those caused by defective development or by excessive development. Abnormal development of the left lobe can lead to gastric volvulus, whereas the right lobe may be asymptomatic, or it may lead to portal hypertension. Anomalies related to excessive development result in the formation of an accessory lobe, which may undergo torsion [9]. The accessory liver tissue may be formed by displacing the primitive, rudimentary organ or persistence of the mesodermal septa during proliferation [10]. A mini-accessory lobe on preoperative imaging might be mistaken for a lesser omental lymphadenopathy, leading to diagnostic confusion [11].

Fissures and furrows can normally form by the invagination of the peritoneum, whereas accessory fissures are formed by anatomic variation and furrows by diaphragmatic indentation [12]. When accumulations in the fissure become loculated, they are mistaken for a cyst, intrahepatic hematoma, or liver abscess. When tumor cells are disseminated and deposited in these fissures, they are mistaken for intrahepatic focal lesions [13]. Pseudolesions present with different shapes and in locations similar to parenchymatous lesions, so knowledge about these lesions is important to avoid misdiagnosis.

In this case, the appearance of the stomach mucosa was normal with multiple rugae on the surface, and a constricting band was visible externally on the posterior surface, extending to the anterior surface, so this anomaly was considered a congenital variation. Typically, a chronic peptic ulcer secondarily leads to fibrosis and constriction of the stomach, the most common etiology for an hourglass constriction. Any differential growth or anomalous attachment of the peritoneum may have led to variation in the morphology of the stomach and liver in this case.

## Conclusions

Although common findings, these variations are clinically important. Documentation of these variations can help surgeons and gastroenterologists correlate diagnosis and treatment and can also help radiologists avoid misdiagnosis during imaging. Moreover, knowledge of these variations can help prevent iatrogenic injuries during laparoscopic surgeries.
